# Supplementary material for: A mathematical model of Bacteroides thetaiotaomicron, Methanobrevibacter smithii, and Eubacterium rectale interactions in the human gut
Source: Sci Rep. 2023 Dec 1;13:21192. doi: 10.1038/s41598-023-48524-4 (PMC10692322; doi:10.1038/s41598-023-48524-4)
Supplement: Supplementary file 1 — Supplementary Information. [file 41598_2023_48524_MOESM1_ESM.pdf]

Supplementary Information for "A mathematical model of *Bacteroides thetaiotaomicron, Methanobrevibacter smithii,* and *Eubacterium rectale* interactions in the human gut"

Melissa A. Adrian[1+*], Bruce P. Ayati[1], and Ashutosh K. Mangalam[2]

[1]University of Iowa, Department of Mathematics, Iowa City, IA, 52242, USA
[2]University of Iowa, Department of Pathology, Iowa City, IA, 52242, USA
[*]maadrian@uchicago.edu
[+]Current Affiliation: University of Chicago, Department of Statistics, Chicago, IL 60637

Appendix A: Data

Longitudinal data on all three species *B. thetaiotaomicron*, *E. rectale*, and *M. smithii* is, to our knowledge, not openly available in the published literature. We were able to extract a single set of data points for our system including all three species and their relevant substrates from Shoaie et al[1].

Given the lack of available longitudinal data for this biological system, we assume that this data, shown in Supplementary Table 1, are the center values of the oscillations for each substrate or biomass quantity. One complication that arises from using this data is that the total biomass for all three species was experimentally measured as a single quantity, which is another factor that further contributes to our uncertainty in our full model parameter estimates. We fit the model parameters to produce numerical solutions with average biomass concentrations that roughly sum to the biomass quantity given in Supplementary Table 1.

Polysaccharides (μM)	32.06
H_2 (μM)	0
CO_2 (μM)	7.96
Acetate (μM)	9.71
Biomass (gDW/L)	0.001412

Supplementary Table 1. Table of experimental data presented in Shoaie et al. 2013[1] of the microorganisms *B. thetaiotaomicron*, *E. rectale*, and *M. smithii* and their substrates CO_2, H_2, acetate, and polysaccharides. The biomass measurement is a combination of the biomass of the three microorganisms.

Appendix B: Optimization and Sensitivity Analysis Details

B.1 Optimization

Our optimization problem involves finding a parameter set $\theta \in \mathbb{R}^{20}$ such that we closely match the steady state solutions of our ODEs in equations (2) and (3) to the data in Supplementary Table 1, where

$$\theta = [\beta_a, \beta_B, \beta_{E_1}, \beta_{E_2}, \beta_{h_1}, \beta_{h_2}, \beta_{h_3}, \beta_{M_1}, \beta_{M_2}, \beta_p, \gamma_a, \gamma_B, \gamma_h, \gamma_p, \mu_{aE}, \mu_{aM}, \mu_{hM}, \mu_{pB}, \mu_{pE}, q].$$

These parameters, though representative of physical quantities, do not have precise estimates in the literature to our knowledge. With a naive or randomly generated initial guess of this parameter set, this optimization will struggle to find a parameter set that produces a steady state solution that closely matches our observations. For this reason, we performed a pre-search of the parameter space to obtain an initial parameter set that produces roughly desireable results. This pre-search was informed by some general principles that we hypothesize are reasonable for our system: chemical reactions happen at a faster timescale compared to microbial replication, and our chemostat proxy of the gut has a slow fluid turnover rate. We list our initialization of θ in Supplementary Table 2.

β_a	3.0×10^5	β_{h_2}	33,000	γ_B	10	μ_{hM}	3,000
β_B	1.2	β_{M_1}	0.827	γ_h	150	μ_{pB}	200,000
β_{E_1}	0.8	β_{M_2}	0.5	γ_p	400	μ_{pE}	5.0×10^6
β_{E_2}	0.6	β_p	1,000	μ_{aE}	50,000	q	0.05
β_{h_1}	400	γ_a	200	μ_{aM}	40,000	β_{h_3}	10,000

Supplementary Table 2. Table of initial values of our parameter set θ for equations (2) and (3).

With this parameter initialization, we performed a constrained optimization of the MAPE score defined in (6) using the Nelder-Mead optimization method. We defined our lower-bound for our constraints on the parameter set to be $[0]^{20}$, and our upper-bound to be $[1e6, 10, 10, 10, 1000, 1e5, 1e5, 10, 10, 1e5, 1e4, 1e4, 1e4, 1e4, 1e6, 1e6, 1e5, 1e8, 1e8, 10]$. The ordering of the parameter values in this vector is consistent with the order shown in θ. Based on this optimization problem, we obtained a final set of parameters shown in Table 2.

B.2 Sensitivity Analysis

We specified our prior distribution of the each of the parameters to follow a Uniform(a_i, b_i) distribution, where we define each a_i and b_i for each parameter in Supplementary Table 3.

The upper and lower bounds were defined to be half and five times, respectively, the parameter estimate from Table 2. This choice of upper and lower bounds for our sensitivity analysis allowed us to remain in a stable region of the parameter space in order to obtain numerically stable ODE solutions for any given parameter sample.

We explored a different specification of each upper and lower bound, where the upper bound was 10 times our parameter estimates from Table 2, and the lower bound was set to 0 to reflect our large uncertainty in our parameter estimation results. This specification of prior distribution resulted in a large number of unstable ODE solutions, and upon inspection, many of these had parameters or sets of related parameters that were extreme or mismatched (*e.g.,* a decay term with ten times greater rate, essentially replacing $\exp(-x)$ with $\exp(-10x)$ for some x, or a large consumption of polysaccharides with low growth of *B. thetaiotaomicron*). This issue was resolved when we narrowed our upper and lower bounds closer to our parameter estimate, but still with significant variation.

We produced first- and total-order Sobol' indices based on 2^{19} ODE solutions from time 0 to 250 hours. In order to obtain a vector output of the ODE solutions to compute the Sobol' indices, we average the ODE solutions beginning from hour 200 until 250, which resulted in a vector in $\mathbb{R}^6$ corresponding to our tracked quantities in our ODE, B, E, M, a, h, and p. We present our first-order Sobol' indices in Figure 6 and our total-order Sobol' indices in Figure 7.

References

1. Shoaie, S. *et al.* Understanding the interactions between bacteria in the human gut through metabolic modeling. *Sci. Reports* **3**, 1–10, DOI: 10.1038/srep02532 (2013).

Parameter	a	b
β_a	160,783	1,607,834
β_B	0.64	6.39
β_{E_1}	0.42	4.24
β_{E_2}	0.26	2.62
β_{h_1}	201	2,060
β_{h_2}	16,286	162,858
β_{h_3}	5,015	50,145
β_{M_1}	0.37	3.75
β_{M_2}	0.23	2.28
β_p	532	5,324
γ_a	119	1,192
γ_B	5.62	56
γ_h	66	663
γ_p	206	2,060
μ_{aE}	24,821	248,240
μ_{aM}	19,520	195,203
μ_{hM}	1,655	16,548
μ_{pB}	170,649	1,706,495
μ_{pE}	2.62e6	2.62e7
q	0.027	0.27

Supplementary Table 3. Table of the lower and upper bounds defining the Uniform(a_i, b_i) prior distributions for $i = 1, \ldots, 20$ indexing the parameter set θ. These distributions were used to produce our sensitivity analysis results in Figures 6 and 7.
